# Option pricing in the moderate deviations regime

**DOI:** 10.1111/mafi.12156

**Published:** 2017-08-25

**Authors:** Peter Friz, Stefan Gerhold, Arpad Pinter

**Affiliations:** ^1^ TU and WIAS Berlin Germany; ^2^ TU Wien Austria

**Keywords:** asymptotics, implied volatility, moderate deviations, option pricing

## Abstract

We consider call option prices close to expiry in diffusion models, in an asymptotic regime (“moderately out of the money”) that interpolates between the well‐studied cases of at‐the‐money and out‐of‐the‐money regimes. First and higher order small‐time moderate deviation estimates of call prices and implied volatilities are obtained. The expansions involve only simple expressions of the model parameters, and we show how to calculate them for generic local and stochastic volatility models. Some numerical computations for the Heston model illustrate the accuracy of our results.

## 1 INTRODUCTION

**Figure 1.1:**
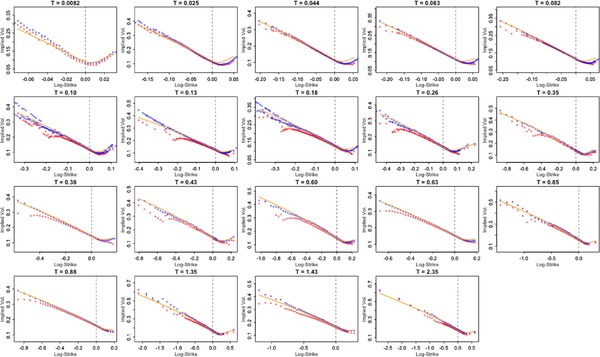
SPX volatility smiles as of August 14, 2013 (courtesy of J. Gatheral). Strikes of options with small remaining time to maturity (T=0.0082) are about $e^{0.02}-1\approx 2\%$ around the money (spot); good data for a later time T=0.26 already have a range of ≈30%. The highest maturity T=2.35 has a range of ≈65% around the money [Color figure can be viewed at http://wileyonlinelibrary.com]

Consider a European call option struck at *K* with remaining time to expiry t>0 and no‐arbitrage price^1^ C(K,t). Today's price of the underlying, the spot value *S*_0_, is known and fixed. Discrete option data are available from the market, typically quoted in (Black–Scholes) implied volatilities; see Figure 1.1. Many option pricing models have been proposed to combine reasonable dynamics for the underlying, small number of parameters and acceptable fits to the data. However, with the notable exception of the Black–Scholes model, closed‐form expressions for call prices are scarce, and approximate pricing formulae have been proposed as substitute: often used to improve calibration, but also toward a better quantitative understanding of a given model. (A classic reference in this context is Gatheral, 2006.)

More specifically, small‐maturity approximations of option prices have been studied extensively in recent years. Starting with Carr and Wu (2003), it was understood that the asymptotic behavior of C(K,t) as t↓0 exhibits very different behavior in the respective cases $K>S_{0}$ (“out‐of‐the‐money”) and $K=S_{0}$ (“at‐the‐money”). We argue that there is a significant asymptotic regime in between, namely,

$$\sqrt{t}\ll K-S_{0}\ll 1.$$

It has received little attention, and, to the best of our knowledge, none at all in the classical diffusion case. The aim of the present paper is to fill this gap. This “moderately out‐of‐the‐money” regime in fact reflects the reality of quoted option prices: As seen in Figure 1.1, the range of strikes tends to concentrate “around‐the‐money” as time to expiry becomes small. At the same time, the regime offers excellent analytic tractability.

To put our results into perspective, we recall some well‐known facts on option price approximations close to expiry. We write c(k,t) for the normalized call price as a function of log‐moneyness $k=\log(K/S_{0})$

(1.1) $$C\left(S_{0}e^{k},t\right)/S_{0}=c\left(k,t\right).$$

In general, c(k,t) depends tacitly on *S*_0_, the (fixed) spot value.^2^ We start with the at‐the‐money (ATM) regime k=0. In the Black–Scholes model, writing c(k,t)=c BS (0,t;σ) with volatility parameter σ>0, we have the following ATM call price behavior

c BS (0,t;σ)∼σt2π,t↓0.

From Muhle‐Karbe and Nutz (2011), the same is actually true in a generic semimartingale model with diffusive component (with spot volatility $\sigma_{0}=\sqrt{v_{0}}>0$),

(1.2) $$c\left(0,t\right)∼\frac{\sigma_{0}\sqrt{t}}{\sqrt{2\pi }},\;t↓0,$$

and this translates to the generic ATM implied variance formula (even in presence of jumps, as long as $v_{0}>0$)

σ imp 2(0,t)=v0+o(1),t↓0.

(We use the notation σ imp (k,t) for the Black–Scholes implied volatility with log‐moneyness *k* and maturity *t*.) Higher order terms in *t* will be model dependent. For instance, in the Heston case, with variance dynamics $dV=-\kappa \left(V-\bar{v}\right)\mathrm{dt}+\eta \sqrt{V}\mathrm{dW}$, implied variance has the ATM expansion

(1.3) σ imp 2(0,t)=v0+a(0)t+o(t),t↓0,a(0)=−η2121−ρ24+v0ρη4+κ2v¯−v0.

This is corollary 4.4 in Forde, Jacquier, and Lee (2012), and we note that *a*(0) has no easy interpretation in terms of the model parameters.

Relaxing k=0 to $k_{t}=o\left(\sqrt{t}\right)$ amounts to what we dub “almost ATM” (AATM) regime.^3^ (In particular, $k_{t}∼t^{\beta }$ is in the AATM regime if and only if β>1/2.) Again for generic semimartingale models with diffusive component and spot volatility $\sigma_{0}>0$, it is easy to see from Caravenna and Corbetta (2016) and Muhle‐Karbe and Nutz (2011) that the ATM asymptotics (1.2) imply the almost ATM asymptotics

$$c\left(k_{t},t\right)∼\frac{\sigma_{0}\sqrt{t}}{\sqrt{2\pi }},\;k_{t}=o\left(\sqrt{t}\right),\;t↓0.$$

This fails when $k_{t}$ ceases to be $o(\sqrt{t})$. Indeed, for $k_{t}=\theta \sqrt{t}$ with constant factor θ>0, we have, from Caravenna and Corbetta (2016) and Muhle‐Karbe and Nutz (2011),

$$c\left(k_{t},t\right)∼E\left[N{\left(-\theta ,\sigma_{0}^{2}\right)}^{+}\right]\sqrt{t},\;t↓0,$$

where $N(-\theta ,\sigma_{0}^{2})$ stands for a Gaussian random variable with mean −θ and variance $\sigma_{0}^{2}$. This, too, holds true in the stated semimartingale generality. In any case, the proof is based on the Lévy case with nonzero diffusity *v*_0_, and the result follows from comparison results, which imply that the difference is negligible to first order. For a thorough discussion of the regime $k=O(\sqrt{t})$ in the (local) diffusion case, see Pagliarani and Pascucci (2017).

Beyond this regime, call price asymptotics change considerably. For instance, take an additional slowly diverging factor log(1/t),

$$k_{t}=\theta \sqrt{t\log\left(1/t\right)}.$$

Even in the Black–Scholes model, we now lose the $\sqrt{t}$‐behavior of call prices described above and in fact

c BS (kt,t;σ)=t12+θ22σ2ℓ(t),

for some slowly varying function ℓ(t), see Mijatović and Tankov (2016). On the other hand, in a genuine out‐of‐the‐money (OTM) situation, with $k_{t}\equiv k>0$ fixed, option values are exponentially small in diffusion models, and we are in the realm of *large deviations theory*. For instance,

c BS k,t;σ=exp−Λ BS (k)t1+o(1),k>0fixed,t↓0,

with Λ BS (k)=12k2/σ2 in the Black–Scholes model. Similar results appear in the literature, with different levels of mathematical rigor, for other and/or generic diffusion models; see Berestycki, Busca, and Florent (2002), Carr and Wu (2003), Forde and Jacquier (2009), and Paulot (2015). Table 1.1 summarizes first‐order call price asymptotics in various models and regimes.

**Table 1.1:** Asymptotic behavior of short‐maturity call options, t↓0

	ATM (At‐the‐Money)	AATM (Almost At‐the‐Money)	MOTM (Moderately Out‐of‐the‐Money)	OTM (Out‐of‐the‐Money)
Process type	$K=S_{0}$	$\log\frac{K}{S_{0}}∼\left(const\right)t^{\beta }$ β>1/2	$\log\frac{K}{S_{0}}∼\left(const\right)t^{\beta }$ 0<β<1/2	$\log\frac{K}{S_{0}}\equiv k>0$
Black–Scholes	$O(\sqrt{t})$, elementary	$O(\sqrt{t})$, elementary	$\exp(-\frac{const}{t^{1-2\beta }})$, elementary	$\exp(-\frac{const}{t})$, elementary
Stochastic or local volatility (diffusion model)	$O(\sqrt{t})$,	$O(\sqrt{t})$,	$\exp(-\frac{const}{t^{1-2\beta }})$	$\exp(-\frac{const}{t})$
Jump diffusion/general semimartingale with diff. component	$O(\sqrt{t})$	$O(\sqrt{t})$	O(t) in finite variation Lévy models^4^	O(t), see Bentata and Cont (2012)

Throughout the paper, we reserve the term OTM for *fixed* OTM log‐strike k>0, to distinguish this regime from the *moderately* out‐of‐the‐money regime that we now define. Our basic observation is that for

(1.4) $$k_{t}∼\left(\mathrm{const}\right)t^{\beta },\;t↓0,$$

the cases of $\beta >\frac{1}{2}$, resp. β=0, are covered by the before‐discussed AATM, resp. OTM, results. This leaves open a significant gap, namely, $\beta \in (0,\frac{1}{2})$, which we call moderately out‐of‐the‐money (MOTM). We have a threefold interest in this MOTM regime,

(1.5) $$k_{t}∼\left(\mathrm{const}\right)t^{\beta },\;t↓0,\;\;\text{for}\;\;\beta \in \left(0,\frac{1}{2}\right).$$

(i) First, it is related to the *reality of quoted (short‐dated) option prices*, where strikes of option price data with acceptable bid–ask spreads tend to accumulate “around the money,” as illustrated in Figure 1.1. To account for this accumulation, we consider strikes that move closer to the money as expiry shrinks, and the simplest way to do so is to consider strikes of the order $k=O(t^{\beta })$ for some β>0. There is no reason why quoted strikes should always be almost ATM (β>1/2), which effectively means an extreme concentration around the money; we are thus led to study the regime (1.5).
(ii) The second reason is *mathematical convenience*. In contrast to the genuine OTM regime (large deviation regime) in which the rate function Λ(k) is notoriously difficult to analyze—often related to geodesic distance problems—MOTM naturally comes with a quadratic rate function and, most remarkably, higher order expansions are always explicitly computable in terms of the model parameters. The terminology *moderately* OTM (MOTM) is in fact in reference to *moderate deviations theory*, which effectively interpolates between the central limit and large deviations regimes.^5^ This also identifies the AATM regime as bordering the central limit regime, where asymptotics are precisely those of the Black–Scholes model, which in turn is the rescaled Gaussian (in log‐coordinates) limit of a general semimartingale model with diffusive component.
(iii) Finally, our third point is that MOTM expansions naturally involve quantities very familiar to practitioners, notably, spot (implied) volatility, implied volatility skew, and so on.

In the Black–Scholes model, it is easy to check that we have the MOTM asymptotics

c BS (kt≡θtβ,t;σ)=exp−θ22σ2t1−2β1+o(1),t↓0.

Loosely speaking, our main results (Theorems 2.3 and 2.5 below) assert that such relations (even of higher order) are true in great generality for diffusion models, and that all quantities are computable and then related to implied volatility expansions. We note in passing that, for Lévy models, the regime (1.5) has been studied by Mijatović and Tankov (2016); then, call prices decay algebraically rather than exponentially. For recent related results on *fractional* stochastic volatility models, see Forde and Zhang (2017) and Guennoun, Jacquier, and Roome (2014). Guillin (2003), who considers small‐noise moderate deviations of diffusions, should also be mentioned here; however, in Guillin (2003), the dynamics depend on a “fast” random environment (with motivation from physics, and no obvious financial interpretation), and the nondegeneracy assumption (D) is not satisfied in our context. The recent related paper by Gao and Wang (2016) contains a *first‐order* moderate deviation principle (MDP) for diffusions under classical regularity assumptions from SDE theory. The main difference to the bulk of our results is that we develop *higher order* expansions, until Section 6 where we revisit first‐order MOTM estimates from a moment‐generating function perspective. However, in this case the underlying models (e.g., Heston) fall immediately outside the scope of Gao and Wang (2016), because of the typical square‐root structure of coefficients. (The matter is discussed in more detail at the beginning of Section 6.)

To round off the introduction, we briefly recall some background on moderate deviations. Consider the classical setting of a centered i.i.d. sequence ${\left(X_{n}\right)}_{n\geq 1}$ with finite exponential moments. Then the empirical means ${\hat{X}}_{n}:=n^{-1}\sum_{k=1}^{n}X_{k}$ converge to zero (law of large numbers, LLN), and this is quantified by an LDP according to Cramér's classical theorem: $P\left[{\hat{X}}_{n}>x\right]=\exp\left(-I\left(x\right)n+o\left(n\right)\right)$ decreases exponentially as n→∞ for fixed x>0, governed by a rate function $I\left(x\right)=\sup_{y\in R}\left(yx-\log E\left[e^{yX_{1}}\right]\right)$. On the other hand, by the CLT, $\sqrt{n}{\hat{X}}_{n}=n^{-1/2}\sum_{k=1}^{n}X_{k}$ has a Gaussian limit law. Moderate deviations cover intermediate scalings, i.e., $\sqrt{na_{n}}{\hat{X}}_{n}$ with $a_{n}\to 0$ and $na_{n}\to \infty$. It turns out (theorem 3.7.1 in Dembo & Zeitouni, 1998) that, for any such sequence $a_{n}>0$, the family $\sqrt{na_{n}}{\hat{X}}_{n}$ satisfies an LDP with speed $1/a_{n}$ and qquadratic rate function. (A natural scaling family is given by $a_{n}=n^{2\beta -1}$, with parameter β>0, so that one considers $\sqrt{na_{n}}{\hat{X}}_{n}=n^{\beta -1}\sum_{k=1}^{n}X_{k}$. Interpolation between LDP, with LLN scaling, and CLT scaling then amounts to considering 0<β<1/2.) This is sometimes called an *MDP*. Formally, an MDP is thus just a certain LDP with appropriate scaling and speed function. Still, the terminology is often useful because of the trichotomy

CLT--MDPforarangeofscalings,withquadraticratefunction--genuineLDP,

which occurs in many situations, not just for i.i.d. sequences of random variables. For references to some other classical moderate deviations results (on empirical measures); e.g., see sections 6.7 and  7.4 of Dembo and Zeitouni (1998). Several authors have investigated moderate deviations in actuarial risk theory; see, e.g., Fu and Shen (2017) and references therein.

The rest of the paper is organized as follows. Section 2 contains our main results, which translate asymptotics for the transition density of the underlying into MOTM call price asymptotics. The corresponding proofs are presented in Section 3. Section 4 and the Appendix give the implied volatility expansion resulting from our call price approximations. Section 5 applies our main results to standard examples, namely, generic local volatility models (Section 5.1), generic stochastic volatility models (Section 5.2), and the Heston model (Section 5.3). As usual, the square‐root degeneracy of the Heston model makes it difficult to apply results for general stochastic volatility models, so we verify the validity of our results—if formally applied to Heston—by a direct “affine” analysis. Finally, in Section 6 we present a second approach to MOTM estimates, which employs the Gärtner–Ellis theorem from large deviations theory. Throughout we take zero rates, a natural simplification in view of our short‐time consideration. Also, w.l.o.g. we normalize spot to $S_{0}=1$.

## 2 MOTM OPTION PRICES VIA DENSITY ASYMPTOTICS

We consider a *general stochastic volatility* model, i.e., a positive martingale ${\left(S_{t}\right)}_{t\geq 0}$ with dynamics

$${\mathrm{dS}}_{t}=S_{t}\sigma_{t}{\mathrm{dW}}_{t},$$

and started (w.l.o.g.) at $S_{0}=1$. We assume that the stochastic volatility process ${\left(\sigma_{t}\right)}_{t\geq 0}$ itself is an Itô‐diffusion, started at some deterministic value σ_0_, called *spot volatility*. Recall that in any such stochastic volatility model, the local (or effective) volatility is defined by

σ loc 2(t,K):=Eσt2|St=K.

As is well known, the equivalent local volatility model

dS∼t=S∼tσ loc (t,S∼t)dWt

has the property that ${\tilde{S}}_{t}=S_{t}$ (in law) for all fixed times. See Brunick and Shreve (2013) for precise technical conditions under which this holds true.^6^ As a consequence, European option prices C(K,t) match in both models. Recall also Dupire's formula in this context

(2.1) σ loc 2(K,t)=∂tC(K,t)12K2∂KKC(K,t),t>0,K>0.

We now state our two crucial conditions.

Assumption 2.1 For all t>0, $S_{t}$ has a continuous pdf K↦q(K,t), which behaves asymptotically as follows for small time:

(2.2) $$q\left(K,t\right)∼e^{-\frac{\Lambda (k)}{t}}t^{-1/2}\gamma \left(k\right),\;t↓0,$$

uniformly for $K=e^{k}$ in some neighborhood of $S_{0}=1$. The energy function Λ is smooth in some neighborhood of zero, with $\Lambda \left(0\right)=\Lambda^{\prime }\left(0\right)=0$. Moreover, $\lim_{k\to 0}\gamma \left(k\right)=\gamma \left(0\right)>0$.

Assumption 2.2 For t↓0 and $K\to S_{0}=1$, the local volatility function of ${\left(S_{t}\right)}_{t\geq 0}$ converges to spot volatility

(2.3) limK→S0t↓0σ loc (K,t)=σ0.

The latter assumption is fairly harmless (in diffusion models; see the beginning of Section 5.2). The first assumption is potentially (very) difficult to check, but fortunately we can rely on substantial recent progress in this direction; see Deuschel, Friz, Jacquier, and Violante (2014a, 2014b) and Osajima (2015). We shall see in Section 5.2 that both assumptions indeed hold in generic stochastic volatility models. Let us also note the fundamental relation between spot volatility σ_0_ (actually equal to implied spot volatility σ_imp_(0, 0) here) and the Hessian of the energy function Λ=Λ(k),

$$\sigma_{0}=\Lambda^{\prime \prime }{\left(0\right)}^{-1/2}.$$

(This is well known [see Durrleman, 2004] and also follows from Proposition 2.4 below.) Now we state our main result. We slightly generalize the log‐strikes considered in (1.5), replacing the constant factor by an arbitrary slowly varying function ℓ.

Theorem 2.3 Under Assumptions 2.1 and 2.2, consider MOTM calls, in the sense that log‐strike is

(2.4) $$k_{t}=t^{\beta }\ell \left(t\right),\;t>0,$$

where ℓ>0 varies slowly at zero and $\beta \in (0,\frac{1}{2})$.

(i) The call price satisfies the moderate deviation estimate
  (2.5) $$\begin{array}{cc} c(k_{t},t) & =\exp\left(-\frac{\Lambda^{\prime \prime }\left(0\right)}{2}\frac{k_{t}^{2}}{t}\left(1+o(1)\right)\right)\\ & =\exp\left(-\frac{1}{2\sigma_{0}^{2}}\frac{k_{t}^{2}}{t}\left(1+o(1)\right)\right),\;t↓0. \end{array}$$
(ii) If we restrict β to $\left(0,\frac{1}{3}\right)$, then the following moderate second‐order expansion holds true

(2.6) $$\begin{array}{cc} c(k_{t},t) & =\exp\left(-\frac{1}{2}\Lambda^{\prime \prime }\left(0\right)\frac{k_{t}^{2}}{t}-\frac{1}{6}\Lambda^{\prime \prime \prime }\left(0\right)\frac{k_{t}^{3}}{t}\left(1+o(1)\right)\right)\\ & =\exp\left(-\frac{1}{2\sigma_{0}^{2}}\frac{k_{t}^{2}}{t}\left(1-\frac{S}{\sigma_{0}^{2}}k_{t}\left(1+o\left(1\right)\right)\right)\right),\;t↓0, \end{array}$$

with spot‐variance $\sigma_{0}^{2}$, equal to σ imp 2(0,0), and implied variance skew S=∂∂k|k=0σ imp 2(k,0).

In particular, for ℓ≡θ>0, we have the (first‐order) expansion

$$t^{1-2\beta }\log c\left(\theta t^{\beta },t\right)∼-\frac{\theta^{2}}{2\sigma_{0}^{2}},\;t↓0,$$

exhibiting a qquadratic rate function $\theta \mapsto \theta^{2}/2\sigma_{0}^{2}$, typical of *moderate deviation problems*.^7^

In a nutshell, (2.5) says that inserting the time‐dependent log‐strike (2.4) into the fixed‐strike OTM/LD approximation c(k,t)=exp(−Λ(k)/t(1+o(1))) yields a correct result, upon Taylor expanding Λ. Mind, however, that this needs a proof using the specifics of our situation, in light of the fact that validity of a large deviation principle does not automatically imply an MDP.

The quantities $\Lambda^{\prime \prime }\left(0\right),\Lambda^{\prime \prime \prime }\left(0\right),\cdots$ appearing above are *always computable* from the initial values and the diffusion coefficients of the stochastic volatility model. This is in stark contrast to the OTM regime, where one needs the function Λ(·), which is in general not available in closed form (with some famous exceptions, like the SABR model). We quote the following result on *N*‐factor models from Osajima (2015) and refer to Section 5.2 for detailed calculations in a two‐factor stochastic volatility model.

Proposition 2.4 Assume that $\left(\log S,\sigma^{1},\cdots ,\sigma^{N-1}\right)$ is Markov, started at $\left(0,{\bar{\sigma }}_{0}\right)$ with ${\bar{\sigma }}_{0}\in R^{N-1}$ and ${\bar{\sigma }}_{0}^{1}>0$, with stochastic volatility $\sigma \equiv \sigma^{1}$, where the generator has (nondegenerate) principal part $\sum a^{\mathrm{ij}}\partial_{\mathrm{ij}}$ in the sense that $a^{-1}$ defines a Riemannian metric. Then

$$\Lambda \left(k\right)=\frac{1}{2b_{1}}k^{2}-\frac{b_{2}}{3b_{1}^{3}}k^{3}+\left(-\frac{b_{3}}{4b_{1}^{4}}+\frac{b_{2}^{2}}{2b_{1}^{5}}\right)k^{4}+O\left(k^{5}\right),\;k\to 0,$$

where the coefficients are given by

$$\begin{array}{cc} b_{1} & =\int_{0}^{1}a^{11}\left(t,{\bar{\sigma }}_{0}\right)\mathrm{dt},\\ b_{2} & =\frac{3}{2}\int_{0}^{1}\left(Va^{11}\right)\left(t,{\bar{\sigma }}_{0}\right)\mathrm{dt},\\ b_{3} & =2\int_{0}^{1}\left(V^{2}a^{11}\right)\left(t,{\bar{\sigma }}_{0}\right)\mathrm{dt}+\frac{1}{2}\int_{0}^{1}\Gamma \left(a^{11},a^{11}\right)\left(t,{\bar{\sigma }}_{0}\right)\mathrm{dt}, \end{array}$$

using the functions

$$\begin{array}{cc} \left(Vf)(t,x\right) & =\sum_{i=1}^{N}a^{1i}\left(t,x\right)\int_{t}^{1}\frac{\partial f}{\partial x_{i}}\left(s,x\right)\mathrm{ds},\\ \Gamma (f,g)(t,x) & =\sum_{i,j=1}^{N}a^{ij}\left(t,x\right)\left(\int_{t}^{1}\frac{\partial f}{\partial x_{i}}\left(s,x\right)\mathrm{ds}\right)\left(\int_{t}^{1}\frac{\partial g}{\partial x_{j}}\left(s,x\right)\mathrm{ds}\right). \end{array}$$

See Osajima (2015, theorem 1(1), with T=1).□

The following result presents a higher order expansion in the MOTM regime. It yields an asymptotically equivalent expression for call prices (and not just logarithmic asymptotics).

Theorem 2.5 Under the assumptions of Theorem 2.3, the logarithm of the call price has the refined MOTM expansion

(2.7) $$\begin{array}{ccc} \log c(k_{t},t) & = & -\sum_{m=2}^{\left\lfloor 1/\beta \right\rfloor }\frac{\Lambda^{\left(m\right)}\left(0\right)}{m!}\frac{k_{t}^{m}}{t}\\ & & +\left(2\beta -\frac{3}{2}\right)\log\frac{1}{t}-2\log\ell \left(t\right)+\log\left(\gamma \left(0\right)v_{0}^{2}\right)+o\left(1\right),\;t↓0. \end{array}$$

This can be expressed equivalently as

$$c\left(k_{t},t\right)∼\gamma \left(0\right)v_{0}^{2}\frac{t^{3/2-2\beta }}{\ell {\left(t\right)}^{2}}\exp\left(-\sum_{m=2}^{\left\lfloor 1/\beta \right\rfloor }\frac{\Lambda^{\left(m\right)}\left(0\right)}{m!}\frac{k_{t}^{m}}{t}\right),\;t↓0.$$

If 1/β is not an integer, then $k_{t}^{m}/t$ tends to infinity for m=⌊1/β⌋, of order $t^{\beta \lfloor 1/\beta \rfloor -1}$ (up to a slowly varying factor). If, on the other hand, 1/β is an integer, then the last summand of the sum $\sum_{m=2}^{\left\lfloor 1/\beta \right\rfloor }$ in (2.7) is of order ℓ(t), which means that the following term log(1/t) may be asymptotically larger. The upper summation limit ⌊1/β⌋ thus ensures that no irrelevant (i.e., *o*(1)) terms are contained in the sum. Note that ⌊1/β⌋=2 for $\beta \in (\frac{1}{3},\frac{1}{2})$, and ⌊1/β⌋≥3 for $\beta \in (0,\frac{1}{3})$, and so (2.7) is consistent with (2.5), resp. (2.6).

The passage from the derivatives of the energy function to ATM derivatives of the implied volatility in the short time limit is best conducted via the BBF formula that was proved in Berestycki et al. (2002). (That said, theses relations are also a direct consequence of our expansions, as is pointed out in Section 4.) In this regard, we have:

Theorem 2.6 Suppose that Λ is a function with the properties required in Assumption 2.1, with $\Lambda^{\prime \prime }\left(0\right)=\sigma_{0}^{-2}=v_{0}^{-1}$, and that the Berestycki–Busca–Florent formula σ imp 2(0,k)=k2/2Λ(k) holds. Then the small‐time ATM implied variance skew and curvature, respectively, relate to Λ via

(2.8) S:=∂∂kk=0σ imp 2(k,0)=−13Λ′′′(0)Λ′′(0)2

and

(2.9) C:=∂2∂k2k=0σ imp 2(k,0)=23Λ′′′(0)2−12Λ′′′′(0)Λ′′(0)3Λ′′(0)3.

By the BBF formula and our smoothness assumptions on Λ,

σ imp 2(k,0)=k22Λ(k)=k2Λ′′(0)k2+13Λ′′′(0)k3+112Λ′′′′(0)k4+O(k5)−1=1Λ′′(0)−13Λ′′′(0)Λ′′(0)2k+19Λ′′′(0)2−112Λ′′′′(0)Λ′′(0)Λ′′(0)3k2+O(k3),k→0.

This implies (2.8) and (2.9).□

Proposition 2.4 combined with Theorem 2.6 allows to compute skew and curvature (and higher derivatives of the implied volatility smile, if desired) directly from the coefficients of a general stochastic volatility model. Related formulae for “general” (even non‐Markovian) models also appear in the work of Durrleman (theorem 3.1.1. in Durrleman, 2004; see also Durrleman, 2010). While not written in the setting of general Markovian diffusion models, and hence not in terms of the energy function Λ, they inevitably give the same results if applied to given parametric stochastic volatility models (see section 3.1 in Durrleman, 2004). However, Durrleman's work comes with some (seemingly) uncheckable assumptions, the drawbacks of which are discussed in section 2.6 of Durrleman (2004).

## 3 PROOFS OF THE MAIN RESULTS

Proof of Theorem 2.3 As the density of $S_{t}$ satisfies $q=\partial_{KK}C$, we have, by Dupire's formula (2.1),

C(K,t)=∫0t∂sC(K,s)ds=∫0t12q(K,s)K2σ loc 2(K,s)ds.

Then, for $K_{t}=e^{k_{t}}$ with $k_{t}↓0$ as stated, we apply Assumption 2.2 as follows:

(3.1) C(Kt,t)=∫0t12q(Kt,s)Kt2σ loc 2(Kt,s)ds∼σ022∫0tq(Kt,s)ds,t↓0.

And then, using local uniformity of our density expansion (2.2), as t↓0,

(3.2) $$\begin{array}{cc} C(K_{t},t) & ∼\frac{\sigma_{0}^{2}\gamma \left(0\right)}{2}\int_{0}^{t}e^{-\frac{\Lambda (k_{t})}{s}}s^{-1/2}\mathrm{dt} \end{array}$$

(3.3) $$\begin{array}{cc} & =\frac{\sigma_{0}^{2}\gamma \left(0\right)}{2}t\int_{0}^{1}e^{-\frac{\Lambda (k_{t})}{xt}}\left(xt\right){}^{-1/2}dx\\ & =\frac{\sigma_{0}^{2}\gamma \left(0\right)}{2}t^{1/2}\int_{0}^{1}e^{-\frac{\Lambda (k_{t})}{xt}}x^{-1/2}dx. \end{array}$$

Because Λ is smooth at zero, and using the fact that $\Lambda \left(0\right)=\Lambda^{\prime }\left(0\right)=0$, we have

$$\frac{\Lambda (k_{t})}{t}∼\frac{1}{2}\Lambda^{\prime \prime }\left(0\right)\frac{k_{t}^{2}}{t}\to \infty \;\text{as}\;t↓0.$$

For small *t*, the integrand in (3.3) is thus concentrated near x=1, and by the Laplace method (theorem 3.7.1 in Olver, 1974)

(3.4) $$\int_{0}^{1}e^{-\frac{\Lambda (k_{t})}{xt}}x{}^{-1/2}dx∼\frac{t}{\Lambda (k_{t})}\exp\left(-\frac{\Lambda (k_{t})}{t}\right),\;t↓0.$$

Therefore,

(3.5) $$\begin{array}{c} C\left(K_{t},t\right)∼\frac{\sigma_{0}^{2}\gamma \left(0\right)}{2}\frac{t^{3/2}}{\Lambda (k_{t})}\exp\left(-\frac{\Lambda (k_{t})}{t}\right)∼v_{0}^{2}\gamma \left(0\right)\frac{t^{3/2}}{\;k_{t}^{2}}\exp\left(-\frac{\Lambda (k_{t})}{t}\right),\;t↓0, \end{array}$$

which implies (recall the notation *c* resp. *C* from (1.1))

(3.6) $$\begin{array}{cc} -\log c(k_{t},t) & =\frac{\Lambda (k_{t})}{t}-\log\frac{t^{3/2}}{k_{t}^{2}}+O\left(1\right)\\ & =\frac{1}{t}\left(\frac{1}{2}\Lambda^{\prime \prime }\left(0\right)k_{t}^{2}+\frac{1}{6}\Lambda^{\prime \prime \prime }\left(0\right)k_{t}^{3}+O\left(k_{t}^{4}\right)\right)+O\left(\log\frac{k_{t}^{2}}{t^{3/2}}\right),\;t↓0. \end{array}$$

To prove (i) and (ii), we thus need to argue that $k_{t}^{2}/t$ dominates $\log(k_{t}^{2}t^{-3/2})$ if $\beta \in (0,\frac{1}{2})$, and that $k_{t}^{3}/t$ dominates $\log(k_{t}^{2}t^{-3/2})$ if $\beta \in (0,\frac{1}{3})$. For m∈{2,3}, we calculate

$$\begin{array}{cc} \frac{k_{t}^{m}/t}{|\log\left(k_{t}^{2}t^{-3/2}\right)|} & =\frac{t^{m\beta -1}\ell {\left(t\right)}^{m}}{\left|\log(t^{2\beta -3/2}\ell {\left(t\right)}^{2})\right|}\\ & =\frac{t^{m\beta -1}\ell {\left(t\right)}^{m}}{|\left(2\beta -\frac{3}{2}\right)\log t+2\log\ell \left(t\right)|}. \end{array}$$

From proposition 1.3.6 (i) in Bingham, Goldie, and Teugels (1987), we know that logℓ(t)=o(logt), and so

$$\frac{k_{t}^{m}/t}{|\log\left(k_{t}^{2}t^{-3/2}\right)|}∼\frac{t^{m\beta -1}\ell {\left(t\right)}^{m}}{|\left(2\beta -\frac{3}{2}\right)\log t|},\;t↓0.$$

This tends to infinity for m=2 and $\beta \in (0,\frac{1}{2})$, and for m=3 and $\beta \in (0,\frac{1}{3})$, as desired.□

Inspecting the preceding proof, it is easy to see that we can expand $\log c(k_{t},t)$ further.

Proof of Theorem 2.5 Taking logs in (3.5) yields

$$\log c\left(k_{t},t\right)=-\frac{\Lambda (k_{t})}{t}+\log\frac{t^{3/2}}{k_{t}^{2}}+\log\left(\gamma \left(0\right)v_{0}^{2}\right)+o\left(1\right),\;t↓0.$$

Then (2.7) follows by Taylor expanding Λ. Note that $k_{t}^{m}/t=o\left(1\right)$ for m≥⌊1/β⌋+1.□

## 4 IMPLIED VOLATILITY

Corollary 4.1 Under the assumptions of Theorem 2.3, let $k_{t}=t^{\beta }\ell \left(t\right)$ with $\beta \in (0,\frac{1}{3})$ and ℓ>0 slowly varying. Then the implied volatility has the MOTM expansion

(4.1) σ imp (kt,t)=σ0−16σ03Λ′′′(0)kt(1+o(1)),t↓0.

We use our main result (Theorem 2.3) in conjunction with a transfer result of Gao and Lee (2014). As the call price tends to zero, we are in case “( − )” of Gao and Lee (2014) (defined on p. 354 of that paper). The notation *L*, *V* of Gao and Lee (2014) means $L=-\log c(k_{t},t)$ resp. V=t1/2σ imp (kt,t), the dimensionless implied volatility. Then corollary 7.2 of Gao and Lee (2014) implies that

(4.2) $$V=\frac{k_{t}}{\sqrt{2L}}\left(1+O(t^{1-2\beta -\varepsilon })\right)+O\left(t^{5/2-4\beta -\varepsilon }\right),\;t↓0.$$

Here, ε>0 denotes an arbitrarily small constant that serves to eat up slowly varying functions in *O*‐estimates (see proposition 1.3.6 (v) in Bingham et al., 1987). By part (ii) of Theorem 2.3, we have

$$2L=\frac{1}{\sigma_{0}^{2}}\frac{k_{t}^{2}}{t}+\frac{\Lambda^{\prime \prime \prime }\left(0\right)}{3}\frac{k_{t}^{3}}{t}\left(1+o\left(1\right)\right),\;t↓0.$$

Inserting this into (4.2) gives

σ imp (kt,t)=t−1/2ktσ0t1/2kt−σ03Λ′′′(0)6t1/21+o(1)+O(t2−4β−ε),t↓0,

which yields (4.1).□

We have no doubt that Corollary 4.1 is true for the whole MOTM regime, i.e., for all $\beta \in (0,\frac{1}{2})$, under very mild assumptions (Assumption A.1 in the Appendix). For any *fixed* $\beta \in (0,\frac{1}{2})$, one can compute the implied volatility expansion using the results of Gao and Lee (2014). However, for β close to $\frac{1}{2}$, more and more terms are needed for the intermediate computations, and there does not seem to be a simple pattern that would allow for a general proof. The details are discussed in the Appendix, where we push the range of β for which (4.1) is proven rigorously to $0<\beta <\frac{3}{7}\approx 0.429$. Note that the expansion in Theorem 2.5 becomes *finer* (i.e., contains more explicit terms) if β is close to zero. Suppose, on the other hand, that β is very close to $\frac{1}{2}$: Then the summands m>⌊1/β⌋=2 in (2.7), which are related to ATM derivatives of implied variance by Theorem 2.6 (see also paragraph (iii) in the Introduction), disappear into the *o*(1)‐term of (2.7).

Corollary 4.1 has some interesting consequences. Under the sheer assumption that implied volatility has a first‐order Taylor expansion for small maturity and small log‐strike of the form

(4.3) σ imp (k,t)=σ0+∂kσ imp (0,0)k+o(k)+O(t),t↓0,k=o(1);

then of course in the MOTM regime, we have $t\ll k_{t}$, and so the *k*‐term dominates the O(t)‐term, which in turn identifies the implied variance skew as

(4.4) S=limt↓02σ0ktσ imp (kt,t)−σ0.

On the other hand, Corollary 4.1 now implies that the right‐hand side of (4.4) equals $-\frac{1}{3}\sigma_{0}^{4}\Lambda^{\prime \prime \prime }\left(0\right)$. We have thus arrived at an alternative proof of the skew representation (2.8) in terms of the energy function, without using the BBF formula. The curvature and higher order derivatives of the ATM smile can be dealt with similarly, if desired.

## 5 EXAMPLES

### 5.1 Generic local volatility models

Clearly, Assumption 2.2 is satisfied for any local volatility model, assuming continuity of the local volatility function. We now discuss Assumption 2.1 and show how to compute our MOTM expansions. First consider the time‐homogeneous local volatility model

(5.1) $${\mathrm{dS}}_{t}=\sigma \left(S_{t}\right)S_{t}{\mathrm{dW}}_{t},\;S_{0}=1,$$

where the deterministic function σ is *C* ^2^ on (0, ∞). An expansion of the pdf q(·,t) of $S_{t}$ has been worked out in Gatheral, Hsu, Laurence, Ouyang, and Wang (2012). They assume growth conditions on σ and its derivatives, which can be alleviated by the principle of not feeling the boundary (appendix A of Gatheral et al., 2012). Proposition 2.1 of Gatheral et al. (2012) states that

(5.2) $$q\left(e^{k},t\right)∼\frac{e^{-k}u_{0}\left(0,k\right)}{\sqrt{2\pi t}}\exp\left(-\frac{\Lambda (k)}{t}\right),\;t↓0,$$

uniformly in *k*, where the energy function is given by (cf. Varadhan, 1967)

$$\Lambda \left(k\right)=\frac{1}{2}{\left(\int_{0}^{k}\frac{dx}{\sigma (e^{x})}\right)}^{2},$$

and

(5.3) $$u_{0}\left(0,k\right)=\sigma {\left(1\right)}^{1/2}\sigma {\left(e^{k}\right)}^{-3/2}e^{-k/2}.$$

(Recall that we normalize spot to $S_{0}=1$ throughout.) This shows that Assumption 2.1 is satisfied, with

(5.4) $$\gamma \left(0\right)=\frac{1}{\sqrt{2\pi }\sigma \left(1\right)}.$$

To evaluate the expansions from Theorem 2.3, we compute the derivatives of Λ

$$\Lambda^{\prime }\left(k\right)=\frac{1}{\sigma (e^{k})}\int_{0}^{k}\frac{dx}{\sigma (e^{x})},\;\Lambda^{\prime \prime }\left(k\right)=\frac{1}{\sigma {\left(e^{k}\right)}^{2}}-\frac{e^{k}\sigma^{\prime }\left(e^{k}\right)}{\sigma {\left(e^{k}\right)}^{2}}\int_{0}^{k}\frac{dx}{\sigma (e^{x})},$$

$$\Lambda^{\prime \prime \prime }\left(k\right)=-\frac{3e^{k}\sigma^{\prime }\left(e^{k}\right)}{\sigma {\left(e^{k}\right)}^{3}}+\left(\frac{2e^{2k}\sigma^{\prime }{\left(e^{k}\right)}^{2}}{\sigma {\left(e^{k}\right)}^{3}}-\frac{\sigma {\left(e^{k}\right)}^{\prime \prime }}{\sigma {\left(e^{k}\right)}^{2}}\right)\int_{0}^{k}\frac{dx}{\sigma (e^{x})},$$

which yield

$$\Lambda^{\prime \prime }\left(0\right)=\frac{1}{\sigma {\left(1\right)}^{2}}=\frac{1}{\sigma {\left(S_{0}\right)}^{2}},$$

(5.5) $$\Lambda^{\prime \prime \prime }\left(0\right)=-\frac{3\sigma^{\prime }\left(1\right)}{\sigma {\left(1\right)}^{3}}=-\frac{3\sigma^{\prime }\left(S_{0}\right)}{\sigma {\left(S_{0}\right)}^{3}}.$$

Alternatively, these expressions can be obtained from Proposition 2.4. As the assumptions of Theorem 2.3 are satisfied, we obtain the following MOTM call price estimates, where $k_{t}=\theta t^{\beta }$ and θ>0

$$\begin{array}{cc} c(k_{t},t) & =\exp\left(-\frac{\theta^{2}}{2\sigma {\left(1\right)}^{2}t^{1-2\beta }}\left(1+o(1)\right)\right),\;\beta \in \left(0,\frac{1}{2}\right),\;t↓0,\\ c(k_{t},t) & =\exp\left(-\frac{\theta^{2}}{2\sigma {\left(1\right)}^{2}t^{1-2\beta }}-\frac{\sigma^{\prime }\left(1\right)}{2\sigma {\left(1\right)}^{3}}\frac{\theta^{3}}{t^{1-3\beta }}\left(1+o(1)\right)\right),\;\beta \in \left(0,\frac{1}{3}\right),\;t↓0. \end{array}$$

Recall from Theorem 2.6 that we denote by S the (limiting small‐time ATM) implied *variance* skew, and so the implied *volatility* skew is given by $S/2\sigma_{0}$, which equals $S/2\sigma \left(1\right)=S/2\sigma \left(S_{0}\right)$ in model (5.1). From (2.8) and (5.5), we find that the *local* skew $\sigma^{\prime }\left(1\right)=\sigma^{\prime }\left(S_{0}\right)$ equals twice the implied volatility skew,

$$\sigma^{\prime }\left(S_{0}\right)=2\times \frac{S}{2\sigma (S_{0})},$$

as observed in remark 5.2 of Henry‐Labordère (2009). Generic time‐inhomogeneous local volatility models

$${\mathrm{dS}}_{t}=\sigma \left(S_{t},t\right)S_{t}{\mathrm{dW}}_{t}$$

can be treated very similarly, using the heat kernel expansion in section 3 of Gatheral et al. (2012), itself taken from Yosida (1953).

### 5.2 Generic stochastic volatility models

We now discuss the results of Section 2 in generic stochastic volatility models. Rigorous conditions under which stochastic volatility models satisfy Assumption 2.1 can be found in Deuschel et al. (2014a) and Osajima (2015). The function Λ is given by the Riemannian metric associated to the model: 2Λ(k) is the squared geodesic distance from $\left(S_{0}=1,\sigma_{0}\right)$ to {(K,σ):σ>0} with $K=e^{k}$. Theorem 2.2 in Berestycki, Busca, and Florent (2004) gives conditions under which Assumption 2.2, concerning convergence of local volatility, is true.

Now we describe how the expressions appearing in the expansions from Theorem 2.3 can be computed explicitly in a generic two‐factor stochastic volatility model

(5.6) $$\begin{array}{cc} {\mathrm{dS}}_{t} & =S_{t}\sqrt{V_{t}}{\mathrm{dW}}_{t},\;S_{0}=1,\\ dV_{t} & =\left(\cdots \right)\mathrm{dt}+\eta \sqrt{V_{t}}\nu \left(V_{t}\right){\mathrm{dZ}}_{t},\;V_{0}=v_{0}>0, \end{array}$$

where ν:R→R and $d{\left\langle W,Z\right\rangle }_{t}=\rho \;\mathrm{dt}$. The Heston model (ν(v)≡1) and the 3/2‐model (ν(v)=v; see Lewis, 2000) are special cases. The infinitesimal generator *L* of the stochastic process (S,V), neglecting first‐order terms, can be written as

$$\begin{array}{c} Lf\approx \frac{1}{2}\mathrm{Tr}\left(\left(\begin{array}{cc} g_{11} & g_{12}\\ g_{21} & g_{22} \end{array}\right)D^{2}f\right),\;f\in C^{2}\left(R^{2}\right), \end{array}$$

where $D^{2}f$ denotes the Hessian matrix of *f*, and the coefficient matrix $g=(g_{ij})$ is given by

$$g=\left(\begin{array}{cc} v & \rho \eta v\nu (v)\\ \rho \eta v\nu (v) & \eta^{2}v\nu {\left(v\right)}^{2} \end{array}\right).$$

We define the constants $b_{1}=g_{11}{|}_{v=v_{0}}=v_{0}$ and $b_{2}=\frac{3}{4}\sum_{i=1}^{2}g_{1i}\partial_{i}g_{11}{|}_{v=v_{0}}=\frac{3}{4}\rho \eta v_{0}\nu \left(v_{0}\right)$. If we assume that the coefficients in (5.6) are nice enough to justify application of the marginal density expansion obtained in Deuschel et al. (2014a) or part (2) of theorem 1 in Osajima (2015), then we get the desired small‐time density expansion (2.2). Moreover, thanks to Proposition 2.4,

$$\Lambda \left(k\right)=\frac{1}{2b_{1}}k^{2}-\frac{b_{2}}{3b_{1}^{3}}k^{3}+O\left(k^{4}\right)$$

as k→0. Therefore, the quantities $\Lambda^{\prime \prime }\left(0\right)=v_{0}^{-1}=\sigma_{0}^{-2}$ and $\Lambda^{\prime \prime \prime }\left(0\right)=-\frac{3}{2}\rho \eta \nu \left(v_{0}\right)/v_{0}^{2}$ can easily be computed, as well as the small‐time ATM implied variance skew

$$S=-\frac{v_{0}^{2}}{3}\Lambda^{\prime \prime \prime }\left(0\right)=\frac{\rho \eta }{2}\nu \left(v_{0}\right).$$

Thus, all quantities appearing in our expansions (Theorem 2.3, Corollary 4.1) have very simple expressions in terms of the model parameters.

### 5.3 The Heston model

This section contains an application of the results of Sections 2 and 4 to the familiar case of the Heston model, where many explicit “affine” computations are possible. At the beginning of Section 5.2, we recalled some general results implying our Assumptions 2.1 and 2.2. The Heston model is not covered by these results, but satisfies Assumptions 2.1 and 2.2 nevertheless, and thus Theorems 2.3 and 2.5 are applicable to Heston. We will explain how both assumptions can be verified rigorously by a dedicated analysis; full details would involve rather dull repetition of arguments that are found in the literature in a very similar form, and are therefore omitted. The model dynamics are

$$\begin{array}{cc} {\mathrm{dS}}_{t} & =S_{t}\sqrt{V_{t}}{\mathrm{dW}}_{t},\;S_{0}=1,\\ dV_{t} & =-\kappa \left(V-\bar{v}\right)\mathrm{dt}+\eta \sqrt{V_{t}}{\mathrm{dZ}}_{t},\;V_{0}=v_{0}>0, \end{array}$$

where $\bar{v},\kappa ,\eta >0$, and $d{\left\langle W,Z\right\rangle }_{t}=\rho \;\mathrm{dt}$ with ρ∈(−1,1). According to Forde and Jacquier (2009), the first‐order OTM (large deviations) behavior of the call prices is

(5.7) tlogc He (k,t)∼−Λ He (k),k>0fixed,t↓0,

where Λ_He_ is the (not explicitly available) Legendre transform of

(5.8) $$\begin{array}{cc} \Gamma (p) & =\frac{v_{0}p}{\eta (\bar{\rho }\cot\left(\eta \bar{\rho }p/2\right)-\rho )}=\frac{v_{0}p}{\eta \left(\bar{\rho }\left(\frac{1}{\eta \bar{\rho }p/2}+O\left(p\right)\right)-\rho \right)}=\frac{v_{0}p}{\frac{1}{p/2}-\eta \rho +O\left(p\right)}\\ & =\frac{v_{0}p^{2}/2}{1-p\eta \rho /2+O(p^{2})}=\frac{v_{0}p^{2}}{2}\left(1+p\eta \rho /2+O(p^{2})\right),\;p\to 0. \end{array}$$

(We use the standard notation ${\bar{\rho }}^{2}=1-\rho^{2}$.) This expansion implies

(5.9) $$\Gamma^{\prime \prime }\left(0\right)=v_{0}=\sigma_{0}^{2}.$$

The locally uniform density asymptotics (2.2) hold, as seen from an easy modification of the arguments in Forde et al. (2012). There, the Fourier representation of the call price was analyzed by the saddle point method to obtain a refinement of (5.7). Proceeding analogously for the Fourier representation of the pdf of $S_{t}$, we get the density approximation

q He (ek,t)=e−k2πt∫−∞−ip∗(k)∞−ip∗(k) Re eiku/tφt(−u/t)du=exp−Λ He (k)tU(p∗(k))2πΓ′′(k)t−1/21+o(1),t↓0,

locally uniformly in *k*, where $\varphi_{t}$ is the characteristic function of $X_{t}=\log S_{t}$, and $p^{*}$ and *U* are defined on p. 693 of Forde et al. (2012). (Note that Forde et al., 2012, use the notation $\Lambda ,\Lambda^{*}$ instead of our Γ,Λ He .) From (5.9) and the fact that $U(p^{*}\left(0\right))=U\left(0\right)=1$, we see that the factor γ(k) from (2.2) converges to

(5.10) $$\gamma \left(0\right)=\frac{1}{\sqrt{2\pi }\sigma_{0}},$$

as k→0.

To verify Assumption 2.2 (convergence of local volatility), the Dupire formula (2.1) can be subjected to an analysis similar to De Marco, Friz, and Gerhold (2013) and Friz and Gerhold (2015). More precisely, $\partial_{KK}C\left(K,t\right)$ in the numerator of (2.1) is the pdf of $S_{t}$, the analysis of which we have just described. Virtually the same saddle point approach can be applied to the numerator $\partial_{t}C\left(K,t\right)$, yielding convergence of the quotient to $\sigma_{0}^{2}$.

We now calculate our MOTM asymptotic expansions for the Heston model. The Legendre transform Λ_He_ is given by Λ He (k)=supx{kx−Γ(x)} with maximizer $x^{*}=x^{*}\left(k\right)$. From general facts on Legendre transforms,

Λ He ′′(k)=1Γ′′(x∗(k)).

We have $x^{*}\left(0\right)=0$, which implies

Λ He ′′(0)=1Γ′′(0)=1v0.

From Theorem 2.3, with $k_{t}=\theta t^{\beta }$ and θ>0, we then obtain the MOTM call price estimate

(5.11) c He (kt,t)=exp−θ22v0t1−2β1+o(1),t↓0.

As for the second‐order expansion, from the expansion (5.8) of Γ, we clearly see that

$$\Gamma^{\prime \prime \prime }\left(0\right)=\frac{3}{2}v_{0}\eta \rho .$$

On the other hand, a general Legendre computation gives

Λ He ′′′(k)=−1Γ′′(x∗(k))2Γ′′′(x∗(k))(x∗)′(k)=−(Λ He ′′k)3Γ′′′(x∗(k)).

Therefore,

Λ He ′′′(0)=−32ηρv02,

in accordance with the expression for generic two‐factor models, found in Section 5.2. For $\beta \in (0,\frac{1}{3})$, Theorem 2.3 (ii) thus implies the second‐order expansion

(5.12) c He (kt,t)=exp−θ22v0t1−2β+ηρ4v02θ3t1−3β1+o(1),t↓0.

By Theorem 2.5 and (5.10), we obtain the following refined call price expansions, as t↓0:

(5.13) logc He (kt,t)=−12σ02kt2t+32−2βlogt+logσ032π+o(1),β∈13,12,

(5.14) logc He (kt,t)=−12σ02kt2t+ηρ4v02kt3t+32−2βlogt+logσ032π+o(1),β∈14,13.

From the relation (2.8) between implied variance skew and $\Lambda^{\prime \prime \prime }\left(0\right)$, we get the explicit expression S He =ηρ/2 for the skew. This agrees with Gatheral (2006, p. 35). The implied volatility expansion (4.4) becomes

(5.15) σ imp (kt,t)=σ0+ηρ4σ0kt1+o(1),t↓0.

**Figure 5.1:**
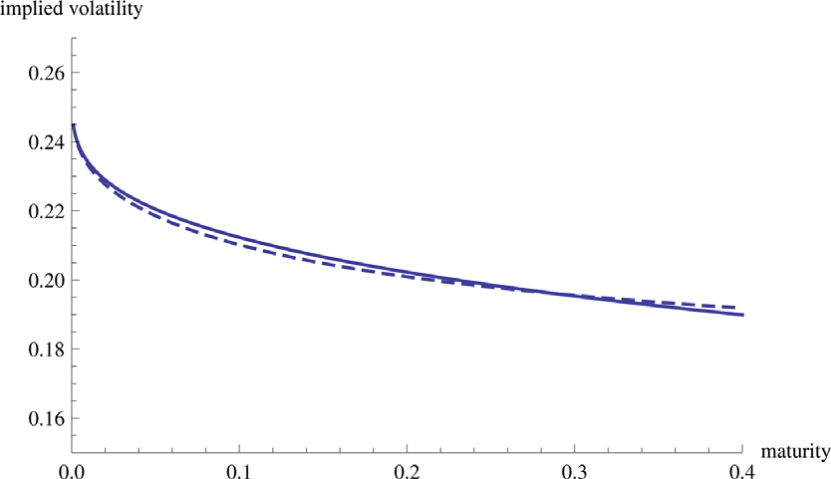
Illustration of our implied volatility expansion for the Heston model, with ℓ≡θ=0.4 and β=0.3. Thus, log‐strike equals $k_{t}=0.4\;t^{0.3}$. The model parameters are $\bar{v}=0.0707$, κ=0.6067, η=0.2928, ρ=−0.7571, $v_{0}=0.0654$ (i.e., $\sigma_{0}=0.2557$), and $S_{0}=1$. The horizontal axis is time. The dashed curve is the exact MOTM implied volatility σ imp (kt,t). The solid curve is the approximation $\sigma_{0}+\frac{\eta \rho }{4\sigma_{0}}k_{t}$ on the right‐hand side of (5.15) [Color figure can be viewed at http://wileyonlinelibrary.com]

Figure 5.1 shows a good fit of this approximation, even for maturities that are not very small.

## 6 OTHER APPROACHES AT MOTM ASYMPTOTICS

In a recent paper, Gao and Wang, 2016 study small noise sample‐path MDPs for SDE solutions, and specialize to the small‐time regime (corollary 4.1.2 in Gao and Wang, 2016). Their asymptotic regime is in fact slightly more general than (2.4), allowing for (in our notation) any $k_{t}$ satisfying $\sqrt{t}\ll k_{t}\ll 1$ as t↓0. (In the financial context, this offers no useful additional flexibility; it allows, e.g., switching between two regimes $k_{t}=t^{\beta_{1}}$ and $k_{t}=t^{\beta_{2}}$ infinitely often as t↓0.) However, Gao and Wang (2016) impose the assumptions of linearly bounded and locally Lipschitz coefficients. These are the typical assumptions for small‐noise LDPs in the literature, but they are rarely satisfied in stochastic volatility models. In particular, their results are not directly applicable to the Heston model. The paper by Cai and Wang (2015) is also worth mentioning here: It presents moderate deviations for the CIR process (the Heston variance process) and a generalization, where the exponent in the dynamics is not necessarily 1/2. The paper uses estimates tied to the (generalized) CIR stochastic differential equation.

In this section, we discuss a different approach at small‐time moderate deviations. While yielding only first‐order results, its conditions are usually easy to check for models with explicit characteristic function. Assumptions 2.1 and 2.2 are not in force here. Recall that in the classical setting of sequences of i.i.d. random variables, a moderate deviation analogue of Cramér's theorem can be deduced by applying the Gärtner–Ellis theorem to an appropriately rescaled sequence (see Dembo & Zeitouni 1998, section 3.7). The MD short‐time behavior of diffusions can be subjected to a similar analysis. Consider the log‐price $X_{t}=\log S_{t}$ with $X_{0}=0$ and mgf (moment‐generating function)

(6.1) $$M\left(p,t\right):=E\left[e^{pX_{t}}\right].$$

Assumption 6.1 For all $\beta \in (0,\frac{1}{2})$, the rescaled mgf satisfies

(6.2) $$\lim_{t↓0}t^{1-2\beta }\log M\left(t^{\beta -1}p,t\right)=\frac{1}{2}\sigma_{0}^{2}p^{2},\;p\in R.$$

We expect that this assumption holds for diffusion models in considerable generality. It is easy to check that (6.2) holds for the Heston model, either by its explicit characteristic function, or, more elegantly, from the associated Riccati equations; see Pinter (2017) for details. Thus, the results of the present section provide an alternative proof of the first order MOTM behavior (5.11) of Heston call prices.

Heuristically, Assumption 6.1 can be derived from the density asymptotics in Assumption 2.1, which in turn hold in quite general diffusion settings (see Deuschel et al., 2014a, 2014b).

(6.3) $$\begin{array}{c} M\left(t^{\beta -1}p,t\right)=\int e^{t^{\beta -1}px}q\left(x,t\right)dx\approx \int \exp\left(t^{\beta -1}px-\frac{\Lambda (x)}{t}\right)dx \end{array}$$

(6.4) $$\begin{array}{cc} & \approx \int \exp\left(t^{\beta -1}px-\frac{\Lambda^{\prime \prime }\left(0\right)x^{2}}{2t}\right)dx \end{array}$$

(6.5) $$\begin{array}{cc} & =\exp\left.{\left(t^{\beta -1}px-\frac{x^{2}}{2\sigma_{0}^{2}t}\right|}_{x=\sigma_{0}^{2}pt^{\beta }}\left(1+o\left(1\right)\right)\right) \end{array}$$

(6.6) $$\begin{array}{cc} & =\exp\left(\frac{1}{2}\sigma_{0}^{2}p^{2}t^{2\beta -1}\left(1+o(1)\right)\right),\;t↓0. \end{array}$$

In (6.3), we ignored that the density expansion (2.2) might not be valid globally in space; this might be made rigorous by estimating q(x,t) by a Freidlin–Wentzell LD argument for *x* sufficiently large. As for (6.4), we can expect concentration near x≈0, because Λ(x) increases with |x|. Finally, (6.5), and thus (6.6), follows from a (rigorous) application of the Laplace method. If (6.6) is correct, then (6.2) clearly follows.

The critical moment of $S_{t}$ is defined by

$$p_{+}\left(t\right):=\sup\left\{p\geq 0:M\left(p,t\right)<\infty \right\}.$$

It is obvious that

(6.7) $$\lim_{t↓0}t^{1-\beta }p_{+}\left(t\right)=\infty$$

is necessary for (6.2); i.e., $p_{+}\left(t\right)$ must grow faster than $t^{\beta -1}$ as t↓0. In the Heston model, e.g., the critical moment is of order $p_{+}\left(t\right)∼\left(\mathrm{const}\right)/t\gg t^{\beta -1}$ for small *t*, as follows from inverting (6.2) in Keller‐Ressel (2011). On the other hand, we do not expect our results to be of much use in the presence of jumps. Indeed, suppose that (6.1) is the mgf of an exponential Lévy model. Then, $p_{+}\left(t\right)\equiv p_{+}$ does not depend on *t*, and is finite for most models used in practice. Therefore, (6.7) cannot hold, and so Assumption 6.1 is not satisfied. The Merton jump diffusion model is one of the few Lévy models of interest that have $p_{+}=\infty$, but it is easy to check that it does not satisfy (6.2), either.

After this discussion of Assumption 6.1, we now give an asymptotic estimate for the distribution function of $X_{t}$ (put differently, MOTM *digital call* prices) in Theorem 6.2. Then, we translate this result to MOTM *call* prices in Theorem 6.3. If desired, higher order terms in (6.2) will give refined asymptotics in Theorem 6.2, using Gulisashvili and Teichmann's (2015) recent refinement of the Gärtner–Ellis theorem. (Further work will be required to translate the resulting expansions into call price asymptotics.) For other asymptotic results on option prices using the Gärtner–Ellis theorem, see, e.g, Forde and Jacquier (2009, 2011).

Theorem 6.2 Under Assumption 6.1 (and without any further assumptions on our model), for $k_{t}=\theta t^{\beta }$ with $\beta \in (0,\frac{1}{2})$ and θ>0, we have a first‐order MD estimate for the cdf of $X_{t}$:

(6.8) $$P\left[X_{t}\geq k_{t}\right]=\exp\left(-\frac{1}{2\sigma_{0}^{2}}\frac{k_{t}^{2}}{t}\left(1+o(1)\right)\right),\;t↓0.$$

Define

$$Z_{t}:=t^{-\beta }X_{t},\;\text{with}\;\text{mgf}\;M_{Z}\left(s,t\right)=E\left[e^{sZ_{t}}\right],$$

and

$$a_{t}:=t^{1-2\beta }=o\left(1\right),\;t↓0.$$

Then, (6.2) is equivalent to

$$\Gamma_{Z}\left(p\right):=\lim_{t↓0}a_{t}\log M_{Z}\left(p/a_{t},t\right)=\frac{1}{2}\sigma_{0}^{2}p^{2},\;p\in R.$$

As $\Gamma_{Z}$ is finite and differentiable on R, the Gärtner–Ellis theorem (theorem 2.3.6 in Dembo & Zeitouni 1998) implies that ${\left(Z_{t}\right)}_{t\geq 0}$ satisfies an LDP as t↓0, with rate $a_{t}$ and good rate function $\Lambda_{Z}$, the Legendre transform of $\Gamma_{Z}$. Trivially, $\Lambda_{Z}$ is qquadratic, too:

$$\begin{array}{cc} \Lambda_{Z}\left(x\right) & =\underset{p\in R}{\sup}\left(px-\Gamma_{Z}\left(p\right)\right)\\ & =\underset{p\in R}{\sup}\left(px-\frac{1}{2}\sigma_{0}^{2}p^{2}\right)=\frac{x^{2}}{2\sigma_{0}^{2}},\;x\in R. \end{array}$$

Now fix θ>0. Applying the lower estimate of the LDP to (θ,∞) yields

$$\begin{array}{cc} \underset{t↓0}{lim inf}a_{t}\log P\left[Z_{t}\geq \theta \right] & \geq \underset{t↓0}{lim inf}a_{t}\log P\left[Z_{t}>\theta \right]\\ & \geq -\Lambda_{Z}\left(\theta \right)=-\frac{\theta^{2}}{2\sigma_{0}^{2}}, \end{array}$$

and applying the upper estimate to [θ,∞) yields

$$\underset{t↓0}{lim sup}a_{t}\log P\left[Z_{t}\geq \theta \right]\leq -\frac{\theta^{2}}{2\sigma_{0}^{2}},$$

and so

$$\lim_{t↓0}a_{t}\log P\left[Z_{t}\geq \theta \right]=-\frac{\theta^{2}}{2\sigma_{0}^{2}}.$$

This is the same as (6.8).□

As in the LD/OTM regime, first‐order cdf asymptotics translate readily into call price asymptotics. The proof of the following result is similar to Pham (2010, p. 30f; concerning the LD regime) and Caravenna and Corbetta (2016, theorem 1.5). In the MD/MOTM regime, one can replace the condition (1.19) of Caravenna and Corbetta (2016) by a mild condition on the moments of the model.

Theorem 6.3 Let $S=e^{X}$ be a continuous positive martingale. Assume that, for all p≥1, its *p*th moment explodes at a positive time (infinity included).^8^ By this we mean that there is a positive $t^{*}\left(p\right)$ such that the mgf $\;E[\exp\left(pX_{t}\right)]$ is finite for all $t\in [0,t^{*}\left(p\right)]$. Let $v_{0}=\sigma_{0}^{2}>0$. Then the following are equivalent

(i) For $k_{t}=\ell \left(t\right)t^{\beta }$, with $\beta \in (0,\frac{1}{2})$ and ℓ>0 slowly varying at zero, it holds that
  $$P\left[X_{t}\geq k_{t}\right]=\exp\left(-\frac{1}{2v_{0}}\frac{k_{t}^{2}}{t}\left(1+o(1)\right)\right),\;t↓0.$$
(ii) Under the assumptions of (i), we have
  (6.9) $$c\left(k_{t},t\right)=\exp\left(-\frac{1}{2v_{0}}\frac{k_{t}^{2}}{t}\left(1+o(1)\right)\right),\;t↓0.$$

First assume (i). Let ε>0 and define ${\tilde{k}}_{t}=\left(1+\varepsilon \right)k_{t}$. Then

(6.10) $$\begin{array}{cc} c(k_{t},t) & \geq E[{\left(e^{X_{t}}-e^{k_{t}}\right)}^{+}\;1_{\left\{X_{t}\geq {\tilde{k}}_{t}\right\}}]\\ & \geq {\left(e^{{\tilde{k}}_{t}}-e^{k_{t}}\right)}^{+}P\left[X_{t}\geq {\tilde{k}}_{t}\right]. \end{array}$$

The first factor is

$${\left(e^{{\tilde{k}}_{t}}-e^{k_{t}}\right)}^{+}={\left({\tilde{k}}_{t}-k_{t}+O\left(k_{t}^{2}\right)\right)}^{+}=\varepsilon k_{t}+O\left(k_{t}^{2}\right),\;t↓0.$$

For the second factor in (6.10), we apply (i) with ${\tilde{k}}_{t}$.

$$\lim_{t↓0}\frac{t}{{\tilde{k}}_{t}^{2}}\log P\left[X_{t}\geq {\tilde{k}}_{t}\right]=-\frac{1}{2v_{0}}.$$

Therefore,

$$\begin{array}{cc} \underset{t↓0}{lim inf}\frac{t}{k_{t}^{2}}\log c\left(k_{t},t\right) & \geq \lim_{t↓0}\frac{t}{k_{t}^{2}}\left(-\frac{1}{2v_{0}}\frac{{\tilde{k}}_{t}^{2}}{t}\left(1+o(1)\right)\right)\\ & =-\frac{{\left(1+\varepsilon \right)}^{2}}{2v_{0}}. \end{array}$$

Now let ε↓0 to get the desired lower bound for $c(k_{t},t)$.

As for the upper bound, we let p>1 and note that, by definition of $p\mapsto t^{*}\left(p\right)$, we have $E[S_{t}^{p+1}]<\infty$ for all $t\in [0,t^{*}\left(p+1\right)]$. Define ${\bar{S}}_{t}=\sup_{0\leq u\leq t}S_{u}$ for t≥0. By Doob's inequality (theorem 3.8 in Karatzas & Shreve, 1991), we have

$$P\left[{\bar{S}}_{t^{*}\left(p+1\right)}\geq s\right]\leq \frac{E\left[S_{t^{*}\left(p+1\right)}^{p+1}\right]}{s^{p+1}},\;s>0.$$

Hence ${\bar{S}}_{t^{*}\left(p+1\right)}$ has a finite pth moment,

$$E\left[{\left({\bar{S}}_{t^{*}\left(p+1\right)}\right)}^{p}\right]=p\int_{0}^{\infty }s^{p-1}P\left[{\bar{S}}_{t^{*}\left(p+1\right)}\geq s\right]\mathrm{ds}<\infty .$$

By the dominated convergence theorem and the continuity of *S*, we thus conclude

(6.11) $$\lim_{t↓0}E\left[S_{t}^{p}\right]=S_{0}^{p}.$$

Now let 1/p+1/q=1 and apply Hölder's inequality.

$$\begin{array}{cc} c(k_{t},t) & =E\left[{\left(e^{X_{t}}-e^{k_{t}}\right)}^{+}\;1_{\left\{X_{t}\geq k_{t}\right\}}\right]\\ & \leq E{\left[{\left({\left(e^{X_{t}}-e^{k_{t}}\right)}^{+}\right)}^{p}\right]}^{1/p}\;P{\left[X_{t}\geq k_{t}\right]}^{1/q}\\ & \leq E{\left[S_{t}^{p}\right]}^{1/p}\;P{\left[X_{t}\geq k_{t}\right]}^{1/q}. \end{array}$$

By (6.11) and (i), we obtain

$$\underset{t↓0}{lim sup}\frac{t}{k_{t}^{2}}\log c\left(k_{t},t\right)\leq \frac{1}{q}\underset{t↓0}{lim sup}\frac{t}{k_{t}^{2}}\log P\left[X_{t}\geq k_{t}\right]=-\frac{1}{2qv_{0}}.$$

Now let p↑∞, i.e., q↓1. The same argument yields the lower bound of the implication (ii) ⟹ (i). The remaining upper bound of (ii) ⟹ (i) is shown very similarly to the lower bound of the implication (i) ⟹ (ii).□

In the light of the general MDP result by Gao and Wang (2016) quoted at the beginning of this section, it might be worth noting that Theorem 6.3 holds, with virtually the same proof, if the assumption $k_{t}=\ell \left(t\right)t^{\beta }$ is replaced by $\sqrt{t}\ll k_{t}\ll 1$.

## ENDNOTES

1 As we focus on stochastic volatility models, which are in general incomplete, it is understood that call prices are computed w.r.t. some fixed pricing measure.

2 There is no spot dependence of the normalized call price in the Black–Scholes model. This holds true, more generally, whenever dynamics for the log‐price $X=\log(S/S_{0})$ are specified without further spot dependence; this includes the Heston model and many other stochastic volatility models.

3 The term “almost ATM” seems new, but this regime was considered by a number of authors including Caravenna and Corbetta (2016) and Muhle‐Karbe and Nutz (2011).

4 This is also true for Lévy models with a Brownian component and a finite variation jump part. For Lévy models with jump part of infinite variation, the call price still decays algebraically, but slower than O(t). See theorem 1 and proposition 2 in Mijatović and Tankov (2016).

5 We write CLT for central limit theorem and LDP for large deviation principle. For readers unfamiliar with moderate deviations, we recall some of the basics toward the end of the Introduction.

6 The situation is very different with jumps; see Friz, Gerhold, and Yor (2014).

7 Recall that the MD rate function for a centered i.i.d. sequence ${\left(X_{n}\right)}_{n\geq 1}$ is given by θ↦θ2/(2 Var (X1)). This is the “moderate” version of Cramér's theorem (theorem 3.7.1 in Dembo and Zeitouni, 1998; see also the Introduction of the present paper).

8 See Keller‐Ressel (2011) for a discussion of moment explosion in affine stochastic volatility models.

## APPENDIX 1 IMPLIED VOLATILITY FOR $\beta \geq \frac{1}{3}$

Assumption A.1 We refine Assumptions 2.1 and 2.2 as follows

(i) Additionally to Assumption 2.1, the relative error in (2.2) is O(t), locally uniformly w.r.t. *k*.
(ii) Convergence of local volatility (see (2.3)) can be refined to
  σ loc (K,t)=σ0+O(t),t↓0,
  uniformly for bounded *K*.
(iii) In (2.2), γ(k) satisfies
  $$\gamma \left(0\right)=\frac{1}{\sqrt{2\pi }\sigma_{0}}.$$

While *proving* Assumption A.1 for stochastic volatility models would go well beyond the scope of the present paper, there are good reasons to believe that it holds in reasonable generality. It does hold for local volatility models, which satisfy (i) according to proposition 2.1 of Gatheral et al. (2012). For stochastic volatility models, the approach of Deuschel et al. (2014a) suggests that the relative error term in (2.2) has a full expansion in (integer) powers of *t*, which would imply (i).

Part (ii) is clear in local volatility models, just assuming smoothness of the local volatility function. In stochastic volatility models, it should be possible to refine the results of Berestycki et al. (2004) (convergence to σ_0_) to a Taylor expansion.

Part (iii) of Assumption A.1 is true for the Heston model (see (5.10)) and generic local volatility models (see (5.4)); the gist of the saddle point argument we applied for Heston, and the resulting expression (5.10), are not tied to that model.
Theorem A.2 Under Assumption A.1, the statement of Corollary 4.1 holds for $\beta \in (0,\frac{3}{7})$.

To simplify notation in the following proof, we write $f{∼}_{\ell }g\left(t\right)$ for two functions *f* and *g*, if f(t)/g(t)∼ℓ(t) as t↓0 for some slowly varying function ℓ. We will use this for functions of algebraic growth order, which are then “almost” asymptotically equivalent. The index ℓ in “${∼}_{\ell }$” is a generic symbol and does not stand for any concrete slowly varying function.

Proof of Theorem A.2 We start by improving the call price expansion from Theorem 2.5, taking into account the asymptotic errors that were made in obtaining it. By part (ii) of Assumption A.1, the relative error in (3.1) is O(t). Part (ii) of Assumption A.1 shows the same for (3.2). The relative error in (3.5) is $O(k_{t})$, as seen from

$$\Lambda {\left(k_{t}\right)}^{-1}=\frac{2v_{0}}{k_{t}^{2}}\left(1+O\left(k_{t}\right)\right),\;t↓0.$$

The only remaining source of error is the application of the Laplace method in (3.4). Here, it does not suffice to state the relative error; we have to do a little better than in the proof of Theorem 2.5. By the higher order extension of the Laplace method (theorem 3.8.1 in Olver, 1974), and because $\Lambda (k_{t})/t\to \infty$ as t↓0, we have the integral expansion

$$\begin{array}{cc} \int_{0}^{1}\exp\left(-\frac{\Lambda (k_{t})}{tx}\right)x^{-1/2}\;dx & =\int_{1}^{\infty }\exp\left(-\frac{\Lambda (k_{t})}{t}x\right)x^{-3/2}\;dx\\ & =\exp\left(-\frac{\Lambda (k_{t})}{t}\right)\frac{t}{\Lambda (k_{t})}\left(1-\frac{3}{2}\frac{t}{\Lambda (k_{t})}+O\left(\frac{t^{2}}{\Lambda {\left(k_{t}\right)}^{2}}\right)\right), \end{array}$$

with error term $t^{2}/\Lambda {\left(k_{t}\right)}^{2}{∼}_{\ell }\;t^{2(1-2\beta )}$. Therefore, our MOTM call price approximation becomes

$$c\left(k_{t},t\right)=\frac{\gamma \left(0\right)\sigma_{0}^{2}}{2}\frac{t^{3/2}}{\Lambda (k_{t})}\exp\left(-\frac{\Lambda (k_{t})}{t}\right)\left(1-\frac{3}{2}\frac{t}{\Lambda (k_{t})}+O\left(t^{2(1-2\beta )-\varepsilon }\right)\right),$$

where ε>0 is arbitrarily small. The Taylor expansion $\Lambda \left(k\right)=\frac{1}{2}\Lambda^{\prime \prime }\left(0\right)k^{2}+\frac{1}{6}\Lambda^{\prime \prime \prime }\left(0\right)k^{3}+O\left(k^{4}\right)$ implies

$$\begin{array}{cc} L_{t} & :=-\log c\left(k_{t},t\right)=\frac{\Lambda (k_{t})}{t}-\log\left(\frac{\gamma \left(0\right)\sigma_{0}^{2}}{2}\frac{t^{3/2}}{\Lambda (k_{t})}\right)+\frac{3}{2}\frac{t}{\Lambda (k_{t})}+O\left(t^{2(1-2\beta )-\varepsilon }\right)\\ & =\frac{1}{2}\Lambda^{\prime \prime }\left(0\right)\frac{k_{t}^{2}}{t}+\log\left(\frac{k_{t}^{2}}{t^{3/2}\sigma_{0}^{4}\gamma \left(0\right)}\right)+\frac{3}{\Lambda^{\prime \prime }\left(0\right)}\frac{t}{k_{t}^{2}}+\frac{1}{6}\Lambda^{\prime \prime \prime }\left(0\right)\frac{k_{t}^{3}}{t}+O\left(t^{\min\{2(1-2\beta ),\beta \}-\varepsilon }\right). \end{array}$$

We now translate the refined call price expansion to implied volatility asymptotics. In the proof of Corollary 4.1, we used corollary 7.2 of Gao and Lee (2014) to achieve the transfer. This would suffice for the interval $\beta \in (0,\frac{2}{5})$, too, but for the larger interval $\beta \in (0,\frac{3}{7})$ we have to take a closer look at the (arbitrary order) asymptotic machinery developed in Gao and Lee (2014). Any unexplained terminology and notation in what follows is as in Gao and Lee (2014). Using proposition 5.6, lemma 5.8, and example 5.13 of Gao and Lee (2014) yields the following estimates in our MOTM regime:

(A.1) $$\begin{array}{cc} & |G_{-}\left(k,\varphi \left(k,L\right)\right)-V|=O\left(\frac{k}{L^{3/2}}\left(\frac{\Psi }{L^{P}}+\frac{1}{L^{N}}\right)\right), \end{array}$$

(A.2) $$\begin{array}{cc} & |G_{-}\left(k,\varphi \left(k,L\right)\right)-G_{-}\left(k,\varphi \left(k,\hat{L}\right)\right)|=O\left(\frac{k}{L^{1/2}}\frac{|L-\hat{L}|}{L}\right), \end{array}$$

(A.3) $$\begin{array}{cc} & \left|G_{-}\left(k,\varphi \left(k,\hat{L}\right)\right)-\frac{k}{\sqrt{2\varphi (k,\hat{L})+k}}\right|=O\left(\frac{k}{L^{1/2}}\frac{k^{2}}{L^{2}}\right), \end{array}$$

with integers N,P≥1, $L=-\log c(k_{t},t)$, an approximation $\hat{L}$ of *L*, dimensionless implied volatility $V:=t^{1/2}\sigma_{\mathrm{imp}}\left(k_{t},t\right)$, and error estimate Ψ. We suppress the time dependence of *k*, *L*, $\hat{l}$, *V*, and Ψ, in order to keep the notation of Gao and Lee (2014). Note that, in the MOTM regime, k/L→0 as t↓0. We have $k{∼}_{\ell }\;t^{\beta }$ and $L{∼}_{\ell }\;t^{2\beta -1}$. The factor $k/L^{1/2}{∼}_{\ell }\;t^{1/2}$ in (A.1)–(A.3) corresponds to the $t^{1/2}$‐term of the dimensionless implied volatility *V*. The error term $k^{2}/L^{2}{∼}_{\ell }\;t^{2(1-\beta )}$ in (A.3) is of negligible order. Therefore, we have to deal with the iteration scheme error $(\Psi /L^{P}+1/L^{N})/L$ in (A.1) and the approximation error $|L-\hat{L}|/L$ in (A.2). We now define the iteration scheme, following Gao and Lee (2014). The choice N=2 and P=2 suffices for our needs. Define a 2‐ply regular iteration scheme $H:=\{h,\eta_{1},\eta_{2}\}$ via the sublog functions

$$\begin{array}{cc} h(\kappa ,\lambda ) & :=\alpha \left(\kappa ,\lambda \right)-\frac{3}{2\lambda }\\ \eta_{1}\left(\kappa ,\lambda \right) & :=\frac{3}{2\lambda }\\ \eta_{2}\left(\kappa ,\lambda \right) & :=\frac{3}{2}\left(\log\left(1+\frac{\alpha (\kappa ,\lambda )}{\lambda }\right)-\frac{\alpha (\kappa ,\lambda )}{\lambda }\right)+\frac{3}{2\lambda }\left({\left(1+\frac{\alpha (\kappa ,\lambda )}{\lambda }\right)}^{-1}-1\right), \end{array}$$

using the auxiliary function

$$\alpha \left(\kappa ,\lambda \right):=-\frac{3}{2}\log\lambda +\log\left(\frac{\kappa }{4\sqrt{\pi }}\right).$$

The corresponding implied volatility approximation function is given by

$$\varphi_{H}\left(\kappa ,\lambda \right):=\lambda +\alpha \left(\kappa ,\lambda \right)-\frac{3}{2\lambda }\left(\alpha \left(\kappa ,\lambda \right)+1\right).$$

The 2‐residual Ψ of *H* yields a sufficiently small iteration scheme error in (A.1):

(A.4) $$\frac{1}{L}\left(\frac{\Psi }{L^{2}}+\frac{1}{L^{2}}\right){∼}_{\ell }\;t^{\min\{3(1-2\beta ),\beta +2(1-2\beta )\}}.$$

Define $\kappa_{t}:=\frac{1}{2\sigma_{0}^{2}}\frac{k_{t}^{2}}{t}$ for t>0 and the constant $C:=\frac{1}{3}\sigma_{0}^{2}\Lambda^{\prime \prime \prime }\left(0\right)$. By inserting $\Lambda^{\prime \prime }\left(0\right)=\sigma_{0}^{-2}$ and $\gamma \left(0\right)={\left(\sqrt{2\pi }\sigma_{0}\right)}^{-1}$ (see part (iii) of Assumption A.1), we get the approximation

$$\begin{array}{cc} {\hat{L}}_{t} & :=\frac{1}{2\sigma_{0}^{2}}\frac{k_{t}^{2}}{t}+\ell_{0}\left(t\right)+3\sigma_{0}^{2}\frac{t}{k_{t}^{2}}+\frac{1}{6}\Lambda^{\prime \prime \prime }\left(0\right)\frac{k_{t}^{3}}{t}\\ & =\kappa_{t}\left(1+\ell_{0}\left(t\right)\kappa_{t}^{-1}+\frac{3}{2}\kappa_{t}^{-2}+Ck_{t}\right), \end{array}$$

where $\ell_{0}\left(t\right):=\log\left(\sqrt{2\pi }k_{t}^{2}/\left(\sigma_{0}^{3}t^{3/2}\right)\right)$ is slowly varying. Thus, the approximation error in (A.2) is

(A.5) $$\frac{|L-\hat{L}|}{L}{∼}_{\ell }\;t^{\min\{3(1-2\beta ),1-\beta \}}.$$

It remains to put together all ingredients. By (A.1), (A.2), and (A.3), combined with (A.4) and (A.5), the following approximation of the dimensionless implied volatility $V_{t}:=t^{1/2}\sigma_{\mathrm{imp}}\left(k_{t},t\right)$ holds

$$\left|\frac{k_{t}}{\sqrt{2\varphi_{H}\left(k_{t},\hat{L_{t}}\right)+k_{t}}}-V_{t}\right|{∼}_{\ell }\;t^{1/2+\min\{3(1-2\beta ),1-\beta \}}.$$

Further calculations show

(A.6) $$\begin{array}{cc} & \frac{k_{t}}{\sqrt{2\varphi_{H}\left(k_{t},\hat{L_{t}}\right)+k_{t}}}\\ & =\frac{k_{t}}{\sqrt{2{\hat{L}}_{t}}}{\left(1+\frac{k_{t}}{2{\hat{L}}_{t}}+\frac{1}{{\hat{L}}_{t}}\alpha \left(k_{t},{\hat{L}}_{t}\right)-\frac{3}{2{\hat{L}}_{t}^{2}}\left(\alpha \left(k_{t},{\hat{L}}_{t}\right)+1\right)\right)}^{-1/2}\\ & =\frac{k_{t}}{\sqrt{2{\hat{L}}_{t}}}\left(1-\frac{1}{2\hat{L}}\alpha \left(k_{t},\hat{L}\right)+\frac{3}{4{\hat{L}}^{2}}\left(\frac{1}{2}\alpha {\left(k_{t},{\hat{L}}_{t}\right)}^{2}+\alpha \left(k_{t},\hat{L}\right)+1\right)+O\left(t^{\min\{3(1-2\beta ),1-\beta \}-\varepsilon }\right)\right), \end{array}$$

because $k_{t}/{\hat{L}}_{t}{∼}_{\ell }\;t^{1-\beta }$, $1/{\hat{L}}_{t}^{3}{∼}_{\ell }\;t^{3(1-2\beta )}$, and $\alpha \left(k_{t},{\hat{L}}_{t}\right){∼}_{\ell }1$. Expansion of the appearing functions yields

$$\begin{array}{cc} \alpha (k_{t},{\hat{L}}_{t}) & =-\ell_{0}\left(t\right)-\frac{3}{2}\ell_{0}\left(t\right)\kappa_{t}^{-1}+O\left(t^{\min\{2(1-2\beta ),\beta \}-\varepsilon }\right),\\ \alpha {\left(k_{t},{\hat{L}}_{t}\right)}^{2} & =\ell_{0}{\left(t\right)}^{2}+O\left(t^{\min\{1-2\beta ,\beta \}-\varepsilon }\right),\\ \frac{1}{{\hat{L}}_{t}} & =\kappa_{t}^{-1}-\ell_{0}\left(t\right)\kappa_{t}^{-2}+O\left(t^{\min\{3(1-2\beta ),1-\beta \}-\varepsilon }\right),\\ \frac{1}{{\hat{L}}_{t}^{2}} & =\kappa_{t}^{-2}+O\left(t^{\min\{3(1-2\beta ),\beta +2(1-2\beta )\}-\varepsilon }\right),\\ \frac{1}{\sqrt{2\hat{L}}} & ={\left(2\kappa_{t}\right)}^{-1/2}\left(1-\frac{1}{2}\ell_{0}\left(t\right)\kappa_{t}^{-1}+\frac{3}{8}\ell_{0}{\left(t\right)}^{2}\kappa_{t}^{-2}-\frac{3}{4}\kappa_{t}^{-2}-\frac{1}{2}Ck_{t}+O\left(t^{\min\{3(1-2\beta ),2\beta \}-\varepsilon }\right)\right). \end{array}$$

Putting these formulas back into (A.6), we get

(A.7) $$\begin{array}{cc} \frac{k_{t}}{\sqrt{2\varphi_{H}\left(k_{t},\hat{L_{t}}\right)+k_{t}}} & =\sigma_{0}t^{1/2}\left(1-\frac{1}{2}Ck_{t}+O\left(t^{\min\{3(1-2\beta ),2\beta ,1-\beta \}-\varepsilon }\right)\right)\\ & =t^{1/2}\left(\sigma_{0}-\frac{1}{6}\sigma_{0}^{3}\Lambda^{\prime \prime \prime }\left(0\right)k_{t}\right)+O\left(t^{1/2+\min\{3(1-2\beta ),2\beta ,1-\beta \}-\varepsilon }\right). \end{array}$$

For the second‐order expansion of the implied volatility to be correct, the error term should be negligible compared to $k_{t}$, which amounts to $t^{\min\{3(1-2\beta ),2\beta ,1-\beta \}}=o\left(k_{t}\right)$. This is true if and only if min{3(1−2β),2β,1−β}>β, which is equivalent to our assumption $\beta \in (0,\frac{3}{7})$.□

For larger β, closer to $\frac{1}{2}$, the whole analysis has to be refined. A more precise iteration scheme *H* has to be chosen, so that the iteration scheme error in (A.4) gets smaller. Moreover, a better log‐price approximation ${\hat{L}}_{t}$ has to be taken into account, using even more terms of the Laplace expansion, in order to decrease the approximation error in (A.5). It should thus be possible to reduce the error in (A.7) to

$$O(t^{\min\{n(1-2\beta ),2\beta ,1-\beta \}-\varepsilon }),\;t↓0,$$

where n∈N can be arbitrarily large. In this fashion, for any *fixed* *n*, it should be straightforward to provide a proof of the second‐order approximation (4.1) of the implied volatility for $\beta <\frac{n}{2n+1}$. That is, we have a clear procedure for any n>2. For small *n*, say n=3,4,⋯, this can be implemented by hand, and larger values (say, n=17) are still feasible with the aid of Mathematica or similar software. In practice, as the calculations in each proof will be tied to that specific value of *n*, very large *n* remains out of reach. Here one would need a new idea to provide an argument for general *n*, which would then prove (4.1) for all $\beta \in (0,\frac{1}{2})$. At this moment, despite some effort, the details of such a construction elude us. Still, we believe that Assumption A.1 suffices to treat the whole interval, i.e., that Theorem A.2 holds with $\frac{3}{7}$ replaced by $\frac{1}{2}$.

Note that the paper by Tehranchi (2016), which presents uniform (nonasymptotic) bounds on implied volatility, does not seem to be applicable here: For $\beta >\frac{1}{3}$, the lower bound of proposition 4.6 in Tehranchi (2016) is not tight enough, as it yields a second‐order term that is asymptotically larger than the second‐order term $k_{t}$ in (4.1).
